# Aqueous Supramolecular Binder for High-Performance Lithium–Sulfur Batteries

**DOI:** 10.3390/polym15122599

**Published:** 2023-06-07

**Authors:** Ruliang Liu, Jiaxin Ou, Lijun Xie, Yubing Liang, Xinyi Lai, Zhaoxia Deng, Wei Yin

**Affiliations:** School of Chemistry and Materials Science, Guangdong University of Education, Guangzhou 510303, China; 15602260148@163.com (J.O.); lijunxie@gdei.edu.cn (L.X.); liangyubin1998@163.com (Y.L.); 13798943464@163.com (X.L.); dengzhaoxia@gdei.edu.cn (Z.D.); yinwei@gdei.edu.cn (W.Y.)

**Keywords:** Li–S batteries, binder, biopolymer

## Abstract

Developing an advanced electrode structure is highly important for obtaining lithium sulfur (Li–S) batteries with long life, low cost, and environmental friendliness. Some bottlenecks, such as large volume deformation and environmental pollution caused by the electrode preparation process, are still hindering the practical application of Li–S batteries. In this work, a new water-soluble, green, and environmentally friendly supramolecular binder (HUG) is successfully synthesized by modifying natural biopolymer (guar gum, GG) with HDI-UPy (cyanate containing pyrimidine groups). HUG can effectively resist electrode bulk deformation through a the unique three-dimensional nanonet-structure formed via covalent bonds and multiple hydrogen bonds. In addition, abundant polar groups of HUG have good adsorption properties for polysulfide and can inhibit the shuttle movement of polysulfide ions. Therefore, Li–S cell with HUG exhibits a high reversible capacity of 640 mAh g^−1^ after 200 cycles at 1C with a Coulombic efficiency of 99%.

## 1. Introduction

At present, due to the unsustainable and polluting nature of traditional fossil energy, there is an urgent need to develop efficient green energy systems to replace fossil energy. Renewable energy sources, such as solar energy and wind energy, have variability and intermittency in the conversion of electrical energy. Therefore, batteries are used as energy storage devices to integrate renewable energy into the grid and achieve the daily use of renewable energy [1]. As a secondary battery, lithium–sulfur (Li–S) batteries have attracted much attention due to their high energy density, environmental friendliness, and low price, and are expected to become one of the primary energy storage batteries for new energy sources [2,3].

Although Li–S batteries have a theoretical capacity of up to 1675 mAh g^−1^, several problems hinder the industrialization of Li–S batteries. On the one hand, due to the large density difference between elemental sulfur and discharge product lithium sulfide, positive electrodes have a volume change of about 80% during cycling, resulting in the peeling of active substances, ultimately presenting poor capacity and cycle stability [4]. On the other hand, the discharge product, long-chain polysulfide lithium (Li_2_Sx,4 ≤ x ≤ 8), is easily dissolved in the electrolyte solvent, which not only causes the loss of active substances but also causes lithium sulfide to diffuse on the surface of the lithium anode, ultimately leading to the decline of cycling life and the low rate performance of Li–S batteries [5].

Various strategies have been explored to alleviate the volume expansion and the dissolution of long-chain lithium polysulfides, such as the use porous materials [6,7,8,9], separator modification [10,11,12,13], solid-state electrolytes [14,15], and functional binders [16,17,18,19,20,21]. As a component of the electrode, the binder is one of the key factors required to improve the performance and prolong the service life of batteries [16,22]. The polyvinylidene fluoride (PVDF) binder is widely used in various battery devices, such as lithium ion batteries and lithium sulfur batteries, due to its good electrochemical stability, acceptable adhesion, and good electrolyte wettability. However, due to the limitations of its non-functionalized linear structure, PVDF can only provide weak van der Waals interactions between the sulfur cathode and the collector, and it cannot resist the volume expansion of the electrode during charging and discharging, resulting in a poor cycling performance [23,24]. Therefore, developing a novel binder is very important for the cycling stability of Li–S batteries under high sulfur loading. Recently, many research efforts have been devoted to developing functional polymeric binders. Feng et al. reported a novel aqueous crosslinked chitosan sulfate network (CCSN) based on the aldimine condensation and coordination reactions of boric acid and polyhydric alcohols. Due to the 3D polymer network of the CSSN, Li–S batteries using the CCSN binder exhibits good cycling performance with a low capacity loss (only 0.082% per cycle) at 1.0C for 500 cycles [25]. Yoo et al. developed a multifunctional polymer binder (PVP/XNBR) by combining a commercial binder and elastic rubber. Because of its desirable mechanical properties and polar functional groups (CN, COOH), the PVP/XNBR binder can interact strongly with polysulfide lithium. Li–S cells with the PVP/XNBR polymer binder exhibited a remarkable improvement in battery performance of 300% after 500 cycles at 1C compared with an Li–S cell using a PVP binder [26]. However, most reported binders still require organic solvents for dissolution, resulting in some problems, such as high price, low conductivity, flammability, toxicity, and environmental pollution. Therefore, there is an urgent need for the development of an environmentally friendly and effective binder.

Due to their low cost, nontoxicity, renewability, and environmentally friendly characteristics, natural materials have been explored to achieve superior binders. Akhtar et al. designed a gelatin–polyethylenimine composite (GPC) as functional binder for Li–S batteries to improve the stability of the electrode during charge–discharge process (with a good capacity retention of about 100% at a current density of 1C) [27]. Jeon et al. employed a natural wood-derived polymer (lignosulfonate sodium salt, LSS) as a binder for Li–S batteries, showing superior capacity and cycle retention because of its unique chemical structure [28]. Wang et al. developed a biopolymer network PPG with a three-dimensional (3D) cross-linked structure, based on guar gum, phytic acid, and soy protein isolate. PPG binder-based LSBs exhibit significant promotion in prolonged cycling tests with a 79.7% capacity retention at 1C [29]. Consequently, the rational design of biopolymer binders is one of the most effective strategies for improving the cycling performance of Li–S batteries.

**Figure 1:**
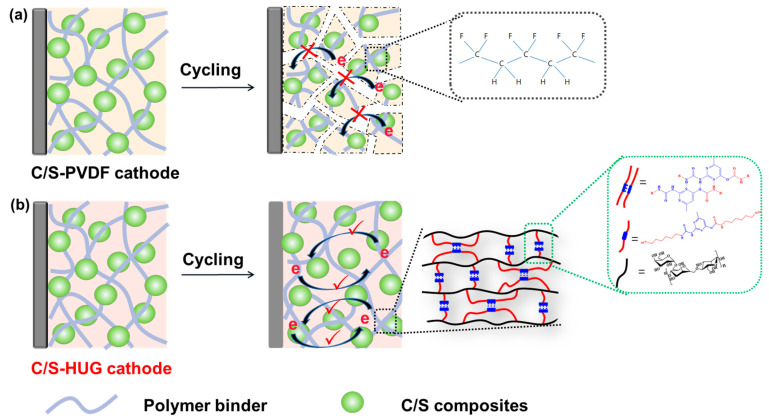
Illustration of the structure of carbon/sulfur composite electrodes with (**a**) PVDF and (**b**) HUG binder before and after cycling. (**a**) Conversional sulfur cathodes with a PVDF binder are prone to cracking and falling off during long cycling. (**b**) The biopolymer supramolecular (HUG) binder with abundant polar groups and multiple hydrogen bonds can maintain a long-lasting electrode structure during long cycling. (note: “×” represents obstructed electronic transmission; “√” represents unobstructed electronic transmission).

In this work, we present a biopolymer binder (HUG) by modifying guar gum (GG) with cyanate containing pyrimidine groups (HDI-UPy), which provides an excellent rate and long-cycling stability in Li–S batteries. Benefiting from good solubility in aqueous solutions, abundant hydrogen bonds, and rich functional groups (carboxyl, imine, hydroxide, and ketone groups), HUG presents good solubility, strong mechanical properties, good self-healing properties, and good LiPS-trapping abilities. As shown in Figure 1, compared with traditional PVDF binder, the HUG binder can provide higher mechanical deformation via robust yet dynamic three-dimensional nanonetworks and stronger lithium polysulfide adsorption via a large number of polar groups. Therefore, Li–S batteries with a HUG binder delivered an initial discharge capacity of 830 mAh g^−1^ and maintained 640 mAh g^−1^ after 200 cycles at 1C.

## 2. Materials and Methods

### 2.1. Materials

Guar gum (GG, Aladdin, Shanghai, China), 2-Amino-6-methyl-4-pyrimidinol (UPy, Aladdin, Shanghai, China), Hexamethylene Diisocyanate (HDI, Aladdin, Shanghai, China), Lithium polysulfide (Li_2_S_6_, Aladdin, Shanghai, China), N,N-dimethylformamide (DMF; Macklin, Shanghai, China), triethylamine (Sigma-Aldrich, St. Louis, MO, USA), ether (Macklin, Shanghai, China), Dibutyltin dilaurate (DBTDL; Macklin, Shanghai, China), n-hexane (Aldrich, Shanghai, China), ethanol (Macklin, Shanghai, China), 1-methyl-2-pyrrolidinone (NMP; Aladdin, Shanghai, China), Super P (Timcal, Shanghai, China), CNT (Aladdin, Shanghai, China), Sulfur (S, St. Louis, MO, USA), and poly(vinylidene fluoride) (PVDF, St. Louis, MO, USA) were used.

### 2.2. Synthesis of HUG Binder

A total of 1.25 g UPy and 16.8 g HDI was added to a 100 mL round-bottomed flask under a nitrogen atmosphere. A small amount of NMP was added to mix, and the mixture was heated at 100 °C for 5 h. Anhydrous chloroform was added to the above mixture for washing under stirring for 2 h, and finally, n-hexane and ether (volume ratio of n-hexane: ether = 6:1) were added for washing, and the crude product was obtained via suction filtration. The obtained crude product was washed three times with diethyl ether and dried under vacuum at 45 °C for 12 h to obtain a white powder (HDI-UPy).

A total of 1 g GG and 0.5 g HDI-UPy was added to a round-bottomed flask with 40 mL DMF under nitrogen atmosphere, and then 0.02 g DBTDL was added to the round-bottomed flask at 90 °C. After condensing and refluxing for 2 h under N_2_ environment, the temperature was raised to 110 °C for 1 h, and then the reaction was halted by lowering the temperature to room temperature. HUG was obtained through filtration. HUG was washed with ethanol several times and dried under vacuum.

### 2.3. Preparation of Cathode and Battery Assembly

CNT and S were mixed with a mass ratio of 2:3, then placed in a vacuum oven at 155 °C for 10 h to obtain a C/S composite. The slurry was fabricated by mixing C/S, Super P, and the binder (HUG, PVDF) at a ratio of 80:10:10. When preparing the slurries containing the HUG and PVDF binder, 1 wt% deionized water and 1 wt% N-methyl-2-pyrrolidone were used as solvents, respectively. The slurry was applied to a carbon-coated aluminum foil. After drying, it was tailored into round disks with a 12 mm diameter. The loading mass of active materials for the electrode plate was approximately 2 mg. CR2032 cells were assembled using a C/S cathode, commercial polypropylene separator (Celgard 2400), and lithium foil anode in an Ar-filled glovebox (MIKROUNA Super), where O_2_ and H_2_O contents were controlled at 1 ppm, and the electrolyte was 1 mol L^−1^ LiTFSI in DME/DOL (1:1) with the addition of 5% FEC.

### 2.4. Characterizations

The morphology and microstructure of the samples were characterized via scanning electron microscope (SEM, TESCAN MIRA LMU, Brno, Czech Republic). The powder X-ray diffraction (PXRD) patterns were obtained using a D8 DISCOVER (Bruker, Billerica, MA, USA) diffractometer at 40 kV and 40 mA using Cu Ka radiation (l = 1.54 Å). Diffraction patterns were recorded over a 2θ angle range within 10° to 60° with a scan rate of 10° min^−1^. Fourier transform infrared spectrometry (FTIR, BRUKERTENSOR27, Bremen, Germany) in the range 4000–400 cm^−1^ were recorded using a Nicolet IS50 infrared analyzer. The thermogravimetric analysis (TGA) of HUG, GG, and C–S was carried out using the TGA 4000 instrument (Thermo Fisher Scientific, Waltham, MA, USA) at a heating rate of 10 °C min^−1^ and in a nitrogen atmosphere. UV–Vis absorption spectra were obtained using an Agilent Cary 5000 UV–Vis–NIR spectrometer (Agilent Technology Co., Ltd., Santa Clara, CA, USA). Cyclic voltammetry (CV) and electrochemical impedance spectroscopy (EIS) were tested using an electrochemical workstation (Coster CS350, Wuhan, China). The CV was scanned at a speed of 0.1 mV s^−1^ (voltage range, 1.6–2.8 V), and EIS was scanned at an AC voltage amplitude of 5 mV between 100 kHz and 0.01 Hz. The galvanostatic charge–discharge test was recorded using the NEWARE Battery Test System (Shenzhen Neware Electronics Co., Ltd., Shenzhen, China) at a voltage range of 1.6–2.8 V (vs. Li/Li^+^) with the calculated C-rate based on the theoretical capacity value of S (1675 mA g^−1^).

## 3. Results and Discussion

**Figure 2:**
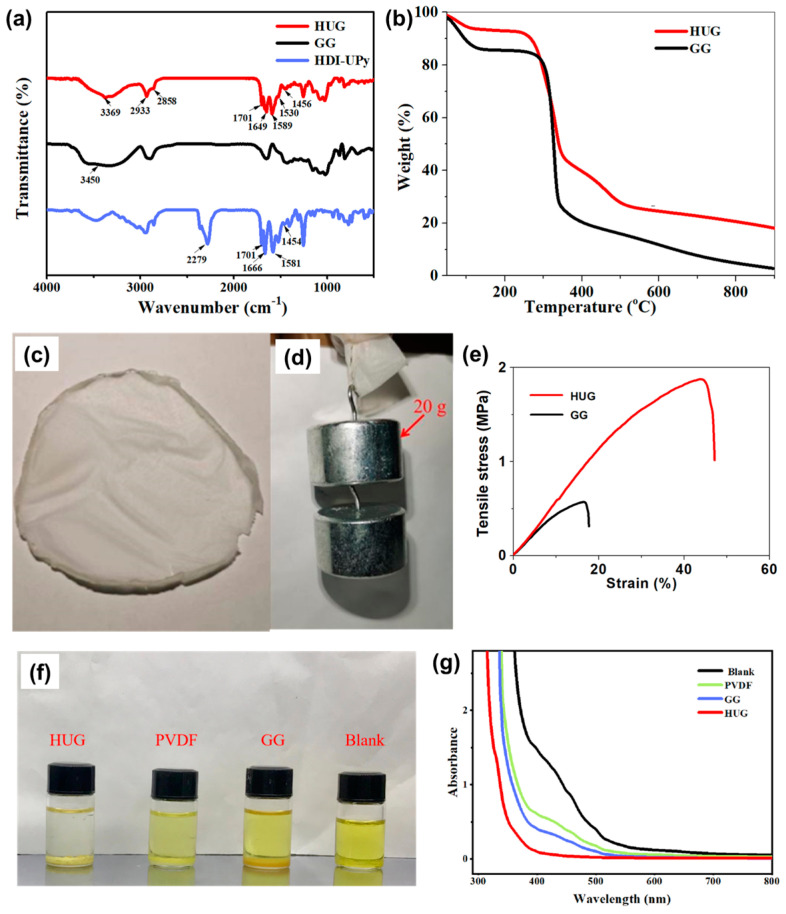
(**a**) Infrared spectra of HDI−UPy, GG, and HUG. (**b**) Thermogravimetric curve of GG and HUG. (**c**) Digital photo of a freestanding HUG membrane. (**d**) Digital photo of HUG withstanding gravity during an experiment. (**e**) Stress–strain curves of GG and HUG. (**f**) Digital image of polysulfide adsorption by PVDF, GG, and HUG in DOL/DME solution. (**g**) UV–Vis spectrum of polysulfide solution before and after adsorption by PVDF, GG, and HUG.

The FTIR spectrum of HDI-UPy, GG, and HUG are shown in Figure 2a. For the spectrum of HDI-UPy, the peaks appearing at 2279, 1666, and 1581 cm^−1^ correspond to the stretching vibration peaks of the cyanate group (-NCO) and the amide group (-CONH-), indicating that HDI-UPy was successfully synthesized [30]. After modifying HDI-UPy on GG, the peak of GG at 3450 cm^−1^ and the cyanate peak of HDI-UPy at 2279 cm^−1^ completely disappeared, while the characteristic peaks of HDI-UPy at 1701, 1649, and 1581 cm^−1^ still existed [31], illustrating that GG and HDI-UPy reacted to synthesize GG–HDI-UPy (HUG). In addition, the peak at 1649 cm^−1^ is the C=O bond extension vibration absorption peak [32]. The peaks at 1456 cm^−1^ and 2933 cm^−1^ are attributed to the bending and stretching vibrations (-CH_2_-) on the main chain, respectively. The peaks that appeared at 3369 cm^−1^ and 1589 cm^−1^ are attributed to the stretching and bending vibrations of the amide (N-H) [29]. These results confirm that the HUG binder was successfully prepared through covalently grafting functional components (HDI–UPy group onto the GG. The thermal stability of GG and HUG was also investigated via thermogravimetric analyses. As shown in Figure 2b, the thermal weight loss of GG is presented in two stages. The first stage is at 149.8 °C with a mass loss of 14.2%, which is caused by the evaporation of crystalline water and free water in GG. The second stage is between 150 and 356 °C with a mass loss of 61.2%. The mass loss in the second stage is mainly caused by the breakage of the structural units in the GG molecular chain as well as the ring structure in the polysaccharide and the breakdown of the polymer backbone. However, the TGA curve of the HIG binder shows a third stage. The first and second stages are both in general agreement with the thermal weight loss process of GG with a mass loss of 49.1%; however, the third stage is caused by the decomposition of HDI-UPy, indicating that the grafting rate of HDI-UPy is about 17.3%. It can thus be concluded that the actual mass ratio of GG to HDI-UPy contained in HDI-UPy–GG is about 3:1, which is close to the mass ratio of GG:HDI-UPy in the synthetic feed at 2:1, indicating that the modification reaction succeeded more completely. In addition, compared to pure GG, the decomposition rate of HUG is significantly lower. Therefore, the introduction of HDI-UPy is beneficial to improving the thermal stability of GG, which is conducive to enhancing the safety of the battery during charging and discharging.

As shown in Figure 2c, a large area flexible HUG membrane can be prepared via simple solvent evaporation, suggesting that HUG has good membrane-forming properties. As an important parameter for maintaining the structural integrity of the electrode, the mechanical properties of a binder are evaluated via the gravity tolerance test and tensile test. A HUG membrane (thickness 0.5 mm) can withstand a weight of 20 g (Figure 2d). On the other hand, the tensile strength and maximum strain are as high as 1.8 MPa and 45.6%, respectively, which is much higher than tensile strength (0.5 MPa) and maximum strain (15.3%) of GG (Figure 2e). The above results indicate that the introduction of multiple hydrogen bonds can form a great number of dynamic cross-linking sites between GG polymer chains, resulting in a tough three-dimensional nano network structure, which is beneficial for enhancing the mechanical properties of GG. In order to investigate the lithium polysulfide adsorption ability of PVDF, GG, and HUG, static lithium polysulfide adsorption tests were carried out in the DOL/DME (1:1) solution containing 1 mM Li_2_S_6_. As displayed in Figure 2f, the colors of different binder-based lithium polysulfide solutions are different.

Compared with the Li_2_S_6_ solutions with PVDF and GG, the Li_2_S_6_ solutions with added HUG are less colored or even colorless, indicating HUG has much stronger adsorption ability than PVDF and GG. Furthermore, ultraviolet–visible (UV–Vis) spectroscopy was also utilized to study polysulfide adsorption on different binders (Figure 2g). The spectrum of pure Li_2_S_6_ solution shows obvious absorption peaks in the region with a wavelength of 400–500 nm. After adding different binders in pure Li_2_S_6_ solution, the absorption peak of UV–Vis spectroscopy begins to decrease to varying degrees. Compared with PVDF and GG, HUG shows the weakest absorption peak intensity in this region, which is consistent with that obtained by visual colorimetry. The excellent lithium polysulfide adsorption ability of HUG is mainly attributed to a plentiful polar group (e.g., N-H, C=O). Its strong adsorption performance can effectively inhibit the shuttle effect of polysulfides, which is expected to improve the utilization rate of cathode materials and enhance the cycle performance of Li–S batteries.

**Figure 3:**
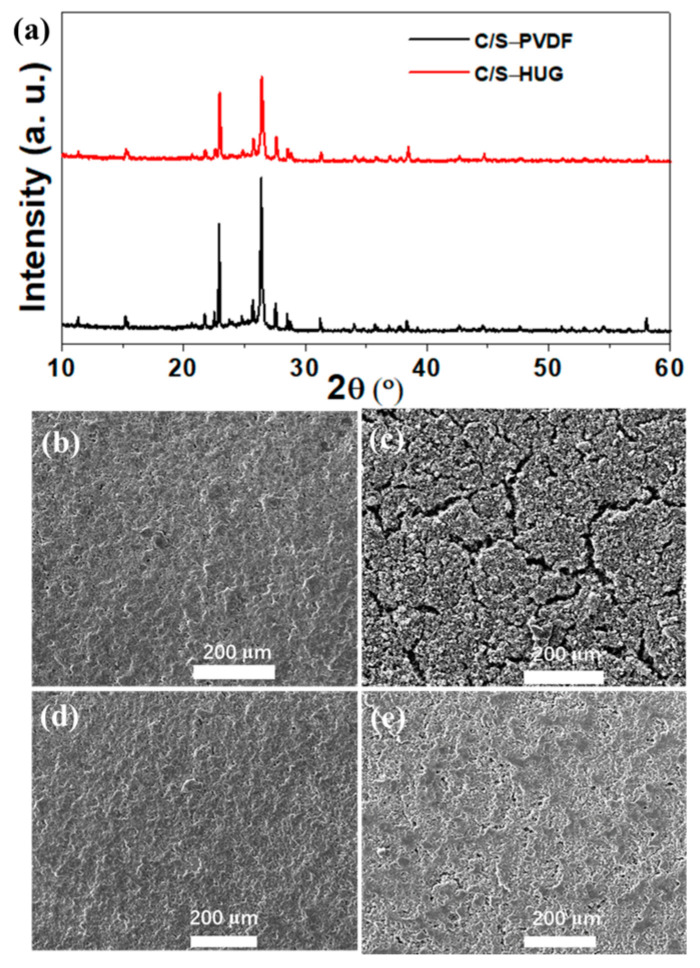
(**a**) XRD curves of C/S–PVDF and C/S–HUG electrode; SEM images of C/S–PVDF (**b**) before and (**c**) after cycling; SEM images of C/S–HUG (**d**) before and (**e**) after cycling.

In order to investigate the effect of binders on the microstructure of electrodes, the XRD characterization of C/S electrodes with HUG and PVDF binders was conducted. As shown in Figure 3a, The XRD test results of the C/S electrode with two binders show that the diffraction peaks at 21.8°, 23.1°, 25.8°, and 26.5° match the (220), (222), (026), and (206) crystal faces of S_8_, respectively, suggesting that S_8_ effectively encapsulates the CNTs tube bundle. The characteristic peaks intensity of the C/S-HUG electrode is significantly lower than that of C/S–PVDF electrode B, indicating that the HUG binder can more effectively promote the dispersion of C/S. Moreover, we also characterize the micromorphology of C/S electrodes with HUG and PVDF binder before and after cycling by means of SEM. As shown in Figure 3b–e, the surface of the original C/S–PVDF electrode shows relatively rough and many pores, while the surface of the original C/S–HUG electrode is relatively smooth and flat, indicating that the HUG binder is more conducive to the uniform dispersion of active materials during the electrode preparation process. After 100 cycles at 1C for Li–S batteries assembled with two kinds of binders, the surface of the C/S–HUG electrode still maintains good structural integrity. In sharp contrast, the cycled C–S@PVDF electrode exhibit many cracks and obvious pulverization, indicating that the PVDF binder can only resist the volume deformation of sulfur electrode during charging and discharging with difficulty. These results illustrate that the HUG binder, with a great number of dynamic cross-linking sites, can significantly enhance the resistance of GG to volume deformation and maintain the integrity of the electrode structure.

**Figure 4:**
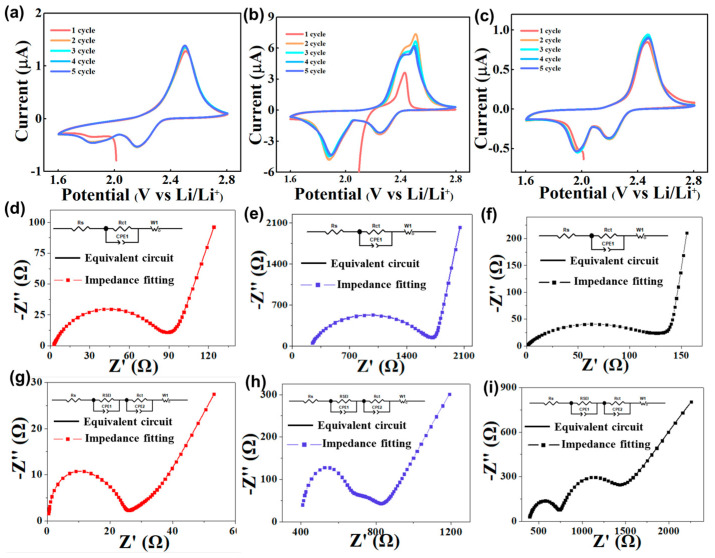
CV curves of Li–S cells with (**a**) HUG, (**b**) GG, and (**c**) PVDF binders; the EIS of Li–S cells with (**d**) HUG, (**e**) PVDF, and (**f**) GG binders before cycling; the EIS of Li–S cells with (**g**) HUG, (**h**) PVDF, and (**i**) GG binders after cycling.

Figure 4a,b shows the CV curves of sulfur cathodes using different binders. Two typical redox peaks at 2.4 V and 2.5 V represent the conversion of elemental sulfur to Li_2_Sx (4 ≤ x ≤ 8) and forward reduced to Li_2_S_2_/Li_2_S with Li_2_Sx. Compared with pure GG binder, the sulfur cathode, using the HUG binder, shows sharper redox peaks and higher peak currents, suggesting that the HUG binder has good sulfur cathode kinetics. In addition, the potential gap between the oxidation peak and the reduction peak of Li–S cells with HUG is 260 mV, which is much smaller than that of Li–S cells with the GG (350 mV) or the PVDF (290 mV) binder, indicating that the HUG binder can greatly decrease the electrochemical polarization of Li–S cells.

To investigate the stability of the electrode interface, the electrochemical impedance spectroscopy (EIS) of Li–S cells with different binders was also tested. Solution resistance (Rs) and charge transfer resistance (Rct) were calculated using fitted EIS data. For Li–S cells without cycling, the Rs and Rct of Li–S cells with HUG are 1.3 and 78.4 Ω, respectively, which is much lower those of Li–S cells with GG (2.3 and 110.5 Ω) and PVDF (221.1 and 1385 Ω) (Figure 4d–f). In Li–S cells after cycling for 100 cycles at a current density of 1C, the Rs and Rct of Li–S cells with HUG decrease to 0.2 and 11.7 Ω. In sharp contrast, the Rct of Li–S cells with GG and HUG is still as high as 554.7 and 184.8 Ω (Figure 4g–i). The results illustrate that the HUG binder can uniformly bond the active material with the conductive agent and fully adhere to the current collector, forming a good conductive network and robust electrode structure, allowing the sulfur electrode to maintain good structural integrity during long-term cycles.

**Figure 5:**
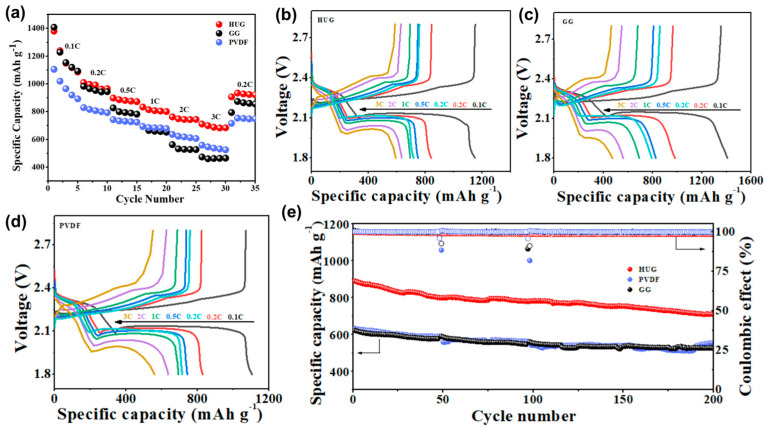
(**a**) Rate performance of Li–S cells with HUG, PVDF, and GG binders; (**b**) the charge–discharge profiles of Li–S batteries with (**b**) HUG, (**c**) GG, and (**d**) PVDF binders at various rates; (**e**) the cycling performance of Li–S batteries based on HUG, PVDF, and GG binders at 1C.

Due to the excellent physical and chemical properties of HUG, we further assemble Li–S cells based on different binders to evaluate the electrochemical performance of the HUG binder. The rate performance was first investigated. As shown in Figure 5a, the initial discharge specific capacities of Li–S cells using the HUG binder at current densities of 0.1C, 0.2C, 0.5C, 1C, 2C, and 3C, respectively, are 1380, 1009, 897, 833, 761, and 710 mAh g^−1^. Finally, when returning to 0.2C, the discharge specific capacity can still reach 880 mAh g^−1^, indicating that the electrode with the HUG binder has good reversibility. Moreover, the specific capacity of Li–S cells using the HUG binder at high current density of 3C (710 mAh g^−1^) is also significantly better than those of Li–S cells using the PVDF binder (559 mAh g^−1^) and the pure GG binder (430 mAh g^−1^), indicating that the HUG binder can improve the rate capability of Li–S batteries. The first discharge and charge profiles of Li–S cells using the HUG binder, PVDF binder, and pure GG binder for various current densities are shown in Figure 5b–d. One charge plateau and two discharge plateaus appear at different current densities, which correspond to the typical redox reaction of lithium polysulfides and the two-step reduction reaction of S_8_, respectively. The second platform of Li–S cells using the HUG binder is longer, indicating that the sulfur has a higher utilization rate. The discharge/charge voltage gap of Li–S cells using different binders is closed at low current density. However, the discharge/charge voltage gap of Li–S cells using the HUG binder is less than 0.3 V when the current density increased to 3C, which is much smaller than that of Li–S cells using the PVDF binder electrode (0.37 V) and the pure GG binder (0.4 V), indicating that the HUG binder can efficiently decrease the polarization and improve the roundtrip energy efficiency.

We also investigated the cycling stabilities of PVDF, GG, and HUG at a high current density of 1C. As shown in Figure 4d, the initial discharge specific capacity of Li–S cells with HUG is 830 mAh g^−1^, while the initial specific capacities of Li–S cells with PVDF and GG are only 633 and 622 mAh g^−1^, respectively. After 200 cycles, the specific discharge capacity of Li–S cells with HUG was still 640 mAh g^−1^, the capacity retention rate was 80% and the average Coulombic efficiency was as high as 99%, indicating that the HUG binder can significantly improve the cycle stability of Li–S batteries at high current density.

**Figure 6:**
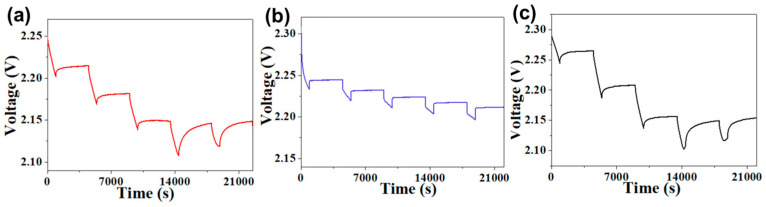
GITT data for Li–S cell with (**a**) HUG, (**b**) PVDF, and (**c**) GG binders.

In order to further investigate the effect of different binders on the electrochemical reaction kinetics in Li–S cells, we estimate the Li^+^ apparent diffusion coefficients (*Ds*) of different electrodes via the galvanostatic intermittent titration technique (GITT) test. The GITT current input consists of discharge pulses at 0.1 mA. Each pulse lasts for 15 min followed by 60 min of rest. The *Ds* of the C–S electrodes with different binders is calculated on basis of the following formula [33]:

(1) $$Ds=(\frac{4}{\pi \tau }){\left(\frac{nV}{S}\right)}^{2}{\left(\frac{{∆E}_{s}}{∆E_{t}}\right)}^{2}$$

where *n* and *V* are the molar mass (mol) and volume (cm^3^) of the active material, respectively; *S* is the cell interfacial area; and τ is the time duration of the pulse. Figure 6 shows the voltage data of discharge pulse from Li–S cells with the HUG, GG, and PVDF binders. The *Ds* of C–S@HUG electrode is 1.37 × 10^−11^ cm^2^ s^−1^, according to Equation (1), which os higher than that of the C–S@GG electrode (1.16 × 10^−11^ cm^2^ s^−1^) and the C–S@PVDF electrode (1.09 × 10^−11^ cm^2^ s^−1^). The GITT test results indicate that the HUG binder can significantly improve the diffusion efficiency of Li^+^ in sulfur electrodes.

## 4. Conclusions

In summary, we developed a new supramolecular binder (HUG) derived from natural biopolymer (GG) for application in Li–S batteries. The good water solubility of the HUG binder eliminates the pollution caused by organic solvents during electrode preparation and greatly reduces the cost of battery preparation. Abundant polar groups in our HUG binder can provide many sites for trapping lithium polysulfide, effectively improving the shuttle effect of polysulfides. Moreover, the HUG binder with plentiful multiple hydrogen bonding can effectively bond the active materials with conductivity additives, forming a good 3D conductive network to accelerate charge transfer, which avoids the damage caused by sulfur cathode volume variations during long-term cycles. Therefore, the HUG binder also delivers a high reversible capacity of 640 mAh g^−1^ after 200 cycles at 1C. This work presents a simple method to prepare an aqueous supramolecular binder based on biopolymers and provides a promising strategy for developing multifunctional binders for high energy density lithium ion batteries.

## Data Availability

Not applicable.
